# Spatiotemporal Distribution of Wine Grape Under Climate Change in Northwestern China

**DOI:** 10.1002/ece3.70826

**Published:** 2025-03-02

**Authors:** Yanyan Liu, Xuejia Shi, Hongjuan Du, Mengjiao Jiang, Fang Li, Jing Wang, Xiaoyu Zhang

**Affiliations:** ^1^ Plateau Atmosphere and Environment Key Laboratory of Sichuan Province, School of Atmospheric Sciences Chengdu University of Information Technology Chengdu Sichuan China; ^2^ Key Laboratory for Meteorological Disaster Monitoring and Early Warning and Risk Management of Characteristic Agriculture in Arid Regions, CMA Yinchuan Ningxia China; ^3^ Yunnan R&D Institute of Natural Disaster on Chengdu University of Information Technology Kunming Yunnan China; ^4^ Wuzhong Meteorological Bureau Wuzhong Ningxia China; ^5^ Ningxia Meteorological Science Institute Yinchuan Ningxia China

**Keywords:** CMIP6 models, MaxEnt, Northwestern China, spatiotemporal distribution, wine grape

## Abstract

The favorable terroir of China's northwest region provides an ideal environment for the cultivation and thriving growth of grapes. However, climate change threatens to alter the optimal grape‐growing regions, presenting considerable challenges to the local wine making industry. To enhance the utilization of regional climate resources and refine the wine grape industry's spatial distribution, this study assessed the performance of nine climate models from the Coupled Model Intercomparison Project Phase 6 (CMIP6) in Northwestern China, simulated the future spatiotemporal distribution of wine grape. Results showed that EC‐Earth3 performed well in simulating temperature, precipitation, and interannual changes. Under the historical periods (1980–2014), the highly suitable areas for wine grapes were predominantly found in the eastern foothills of Helan Mountain in Ningxia, along the Yellow River in Wuhai and Linhe of Inner Mongolia, along the Qilian Mountains in Wuwei, Zhangye and Jiayuguan of Gansu, and along rivers and oases in the northern foothills of Tianshan Mountains, Ili River Valley, Tuha Basin, Yanqi Basin, Aksu, Muzart, and Kashgar of Xinjiang. Compared with historical periods, the highly and moderately suitable areas were expected to expand under SSP245 and SSP585. Nevertheless, the overall pattern of spatial distribution was not anticipated to experience substantial alterations. In the next 50 years (2055–2085), the suitable areas under SSP245 scenario would be higher than SSP585. Precipitation from July to September (*pr*79), soil pH (*ph*), elevation (*dem*), and near‐surface air temperature in January (*tas*1) were the main factors affecting the suitable areas of wine grapes. Further analysis revealed that a certain level of the near‐surface air temperature in January (*tas*1) contributed positively to the expansion of suitable areas. However, excessively high average temperatures in January and July tended to have a detrimental effect. A rise in winter temperature can foster a more favorable environment for wine grapes to overwinter. However, frequent summer heat waves and high winter temperatures caused by climate warming may have adverse effects.

## Graphical abstract

In order to provide guidance for better utilizing local climate resources and optimizing the layout of the wine grape industry, this study compared the applicability of nine climate models of CMIP6 in the Northwestern China, simulated the wine grape suitable areas and predicting their future trends. Results showed that EC‐Earth3 performed well in simulating temperature, precipitation, and interannual changes. Under the historical periods (1980–2014), the highly suitable areas for wine grapes in the northwest region were mainly distributed in the eastern foothills of Helan Mountain in Ningxia, along the Yellow River in Wuhai and Linhe of Inner Mongolia, along the Qilian Mountains in Wuwei, Zhangye, and Jiayuguan of Gansu, and along rivers and oases in the northern foothills of Tianshan Mountains, Ili River Valley, Tuha Basin, Yanqi Basin, Aksu, Muzart, and Kashgar of Xinjiang. Compared with historical periods (1980–2014), the highly and moderately suitable areas of wine grapes under SSP245 and S585 would increased, but the their overall spatial distribution pattern would not undergone significant changes. In the next 50 years (2055–2085), the suitable areas under SSP245 scenario will be higher than SSP585. Precipitation from July to September (pr79), soil pH (ph), elevation (dem), and near‐surface air temperature in January (tas1) were the main factors affecting the suitable areas of wine grapes. Further analysis showed that a certain degree of tas1 was conducive to the increase for suitable areas, but excessively high average temperatures in January and July would have the opposite effect. By comparison, pr79 was the most important factor affecting the suitable areas. An increase in winter temperature can provide a better wintering environment for wine grapes. However, frequent summer heat disasters and high winter temperatures caused by climate warming may have adverse effects.

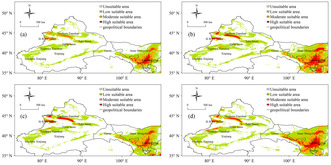

## 1 Introduction

Holding the largest share in both production and consumption within the global fruit wine market (Mu et al. 2022), the grape wine industry possesses substantial market potential and significant added value, thereby making a crucial contribution to regional economic growth (Duchêne 2016). According to the International Vine and Wine Organization (OIV), in 2022, China's grape cultivation area ranked third globally, and its wine production was fourth, indicating the country's significant position in the international fruit wine market. The northwest region, particularly the eastern slopes of the Helan Mountains in Ningxia, has emerged as a key wine grape production area in China due to its favorable hydrothermal conditions, and is recognized as one of the world's most suitable regions for cultivating high‐quality wine grapes (Zhang et al. 2007). The scientific community widely acknowledges the reality of global climate change. The Sixth Assessment Report (AR6) by the Intergovernmental Panel on Climate Change (IPCC) highlights that human activities have exerted a substantial influence on the climate system since the onset of industrialization. Climate is a critical determinant of grape cultivation and quality (Van Leeuwen and Darriet 2016; Honorio, García Martín, and Moral 2018). Consequently, Climate warming is poised to significantly impact the cultivation, phenological development, and suitable growing regions for wine grapes, which in turn will lead to alterations in wine quality (Bernáth et al. 2021; Dinu et al. 2021; Droulia and Charalampopoulos 2021; Zhang and Tang 2021; Feng et al. 2022). Therefore, researching the climate suitability for wine grapes in Northwest China and forecasting shifts in their optimal growing areas is essential. Such studies can facilitate the more effective harnessing of local climate resources, thereby enhancing the high‐quality production of wine grapes, and solidifying the region's advantage position in this industry.

Heat, water, and light are the primary climatic factors influencing grape growth and development (Xu et al. 2011; Yu et al. 2022). Grapevines have stringent heat requirements, with heat indices—such as accumulated temperature, frost‐free period, and high summer temperatures—being determinants in grape cultivation (Wang, Li, and Wang 2017; Wang, Zheng et al. 2017; Yang et al. 2019; Huang et al. 2023). Water conditions are a significant factor in determining grape cultivation zones, especially when considering the overlap of rainy and hot seasons in China (Sun et al. 2013; Li et al. 2023). Precipitation, which is tied to water availability, and dryness bioclimatic indices, which reflect the water balance for plants, are key indicators in this context. The hydrothermal coefficient is a pivotal indicator in grape zoning, representing the ratio of potential evaporation to precipitation, and it provides insight into the region's aridity or humidity (Zhang et al. 2008; Yang et al. 2014). Grapevines are also a light‐loving plants (Feng et al. 2022; Li et al. 2023); thus, sufficient illumination is essential for fostering healthy fruit development, enhancing both yield and the quality of the grapes (Wang et al. 2020).

In recent years, a wealth of scholarly research has been dedicated to examining the distribution of grape production in China. For instance, Mu et al. (2016) employed the Moran's I index to analyze the spatial layout of grape production across the country, synthesizing macro and micro‐level findings. Wang, Li, and Wang (2017) and Wang, Zheng, et al. (2017) conducted comprehensive climate zoning studies for wine grapes in regions such as Sichuan, Xinjiang, and Gansu, offering insightful cultivation recommendations. MaxEnt, a species distribution model founded on the principle of maximum entropy, leverages species distribution data and environmental data to forecast suitable habitats for species. Zhang et al. (2021) utilized MaxEnt to simulate the potential distribution and climatic characteristics of table grapes in Sichuan Province. Similarly, Lin et al. (2020) employed MaxEnt to evaluated the spatial distribution of Cabernet Sauvignon in Hengduanshan valley, Western Sichuan. Building on these studies, our research compared the applicability of nine climate models in Northwestern China. We used MaxEnt to predict the current and future geographical distribution of wine grapes and analyzed the environmental factors influencing their adaptability. The findings offer valuable scientific insight for the strategic introduction, large‐scale cultivation, and optimal spatial planning of wine grapes in the northwest region.

## 2 Materials and Methods

### 2.1 Study Area and Data Sources

**FIGURE 1:**
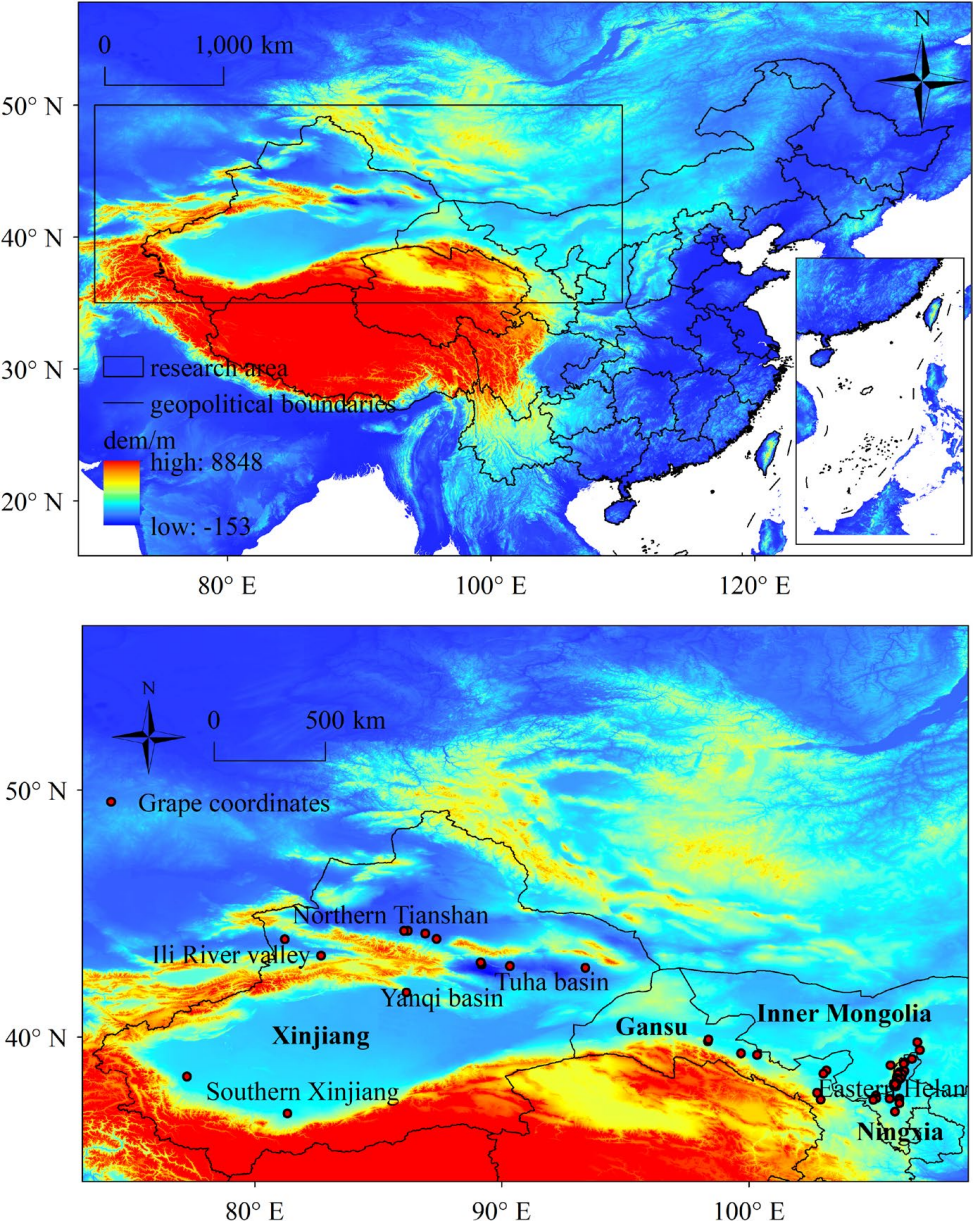
Distribution of wine grape cultivation sites in the Northwestern China.

The study focused on China's northwest region, with a spatial scope defined by the latitudes 35° N to 55° N and longitude 70° E to 110° E. The altitude within this area varies significantly, from a low of −153 m at Aiding Lake in the Turpan Basin to a high of 8848 m at Mount Everest in the Himalayas (Figure 1). We selected the main wine grape varieties cultivated in the region, including Cabernet Sauvignon, Merlot, Pinot Noir, and Chardonnay. Coordinate data were collated from viticultural area records and relevant scientific literature. To enhance the precision of MaxEnt model, the coordinate data underwent a de‐autocorrelation process. The resultant dataset predominantly encompasses localities in the eastern foothills of the Helan Mountains in Ningxia (28), Wuhai and Alxa League in Inner Mongolia (3), Wuwei and Zhangye in Gansu (8), the northern foothills of Tianshan Mountain in Xinjiang, the Ili River Valley, the Tuha Basin, as well as three prefectures in Yanqi Basin and southern Xinjiang (13), summing to a total of 52 coordinate points.

Historical daily meteorological data, comprising average temperature (*tas*), maximum temperature (*tasmax*), minimum temperature (*tasmin*), precipitation (*pr*), average wind speed (*sfcWind*), and relative humidity (*hurs*), were extracted from the CN05.1 grid observation dataset. Hourly ground pressure (*ps*), net solar radiation (*ssr*), and net heat radiation (*str*) were sourced from the ERA5 (cds. climate. copernicus. eu) dataset. The temporal scope of the dataset spans from January 1, 1980, to December 31, 2014, with a spatial coverage extending from 30° N to 55° N latitude and 60° E to 110° E longitude, and a horizontal resolution of 0.25° × 0.25°.

Future daily climate projections were derived from the Earth System Grid Federation (ESGF, https://aims2.llnl.gov), and 9 CMIP6 global circulation model datasets were selected (Table 1). For comparative purposes, shared socioeconomic pathway (SSP)245 and SSP585 were designated as the future climate change scenarios. Under SSP245, the additional radiative forcing was projected to reach 4.5 W/m^2^ by 2100, resulting in an approximate increase in the global average surface temperature of 2.6°C. Precipitation patterns are anticipated to increase by 13%–17%, with notable variations in temperature fluctuations across seasons, summer and spring were expected to exhibit an increasing trend, whereas winter and autumn are projected to show a decreasing trend. Under the SSP585 scenario, the additional radiative forcing by 2100 was expected to escalate to 8.5 W/m^2^, with a more pronounced global warming effect, potentially leading to an increase in the frequency and intensity of extreme weather events and exacerbating drought conditions. Under SSP585, it was projected that by 2100, the global average surface temperature would rise by more than 4.5°C, which could have significant and far‐reaching implications for the global climate system (Yang et al. 2021; Zhang et al. 2022). Given the disparity in horizontal resolution across different modes, this study employed bilinear interpolation to standardize all model data to a uniform horizontal resolution of 0.5° × 0.5°to facilitate comparative analysis. The variables under consideration encompass near‐surface air temperature (*tas*), daily maximum near‐surface air temperature (*tasmax*), daily minimum near‐surface air temperature (*tasmin*), precipitation (*pr*), air pressure (*ps*), near‐surface wind speed (*sfcWind*), near‐surface relative humidity (*hurs*), surface downwelling longwave radiation (*rlds*), surface upwelling long wave radiation (*rlus*), surface downwelling shortwave radiation (*rsds*), and surface upwelling shortwave radiation (*rsus*). The temporal scope spans from January 1, 1980, to December 31, 2085, and is segmented into 1980–2014 period, 2035–2065 period, and 2055–2085 period.

**TABLE 1:** Basic information of the 9 CMIP6 global climate models used in this study.

Model name	Country or regions	Organization	Resolution ratio	Reference
ACCESS‐ESM1‐5	Australia	Commonwealth Scientific and Industrial Research Organization	192 × 144	Mackallah, Chamberlain, and Law (2022)
BCC‐CSM2‐MR	China	Beijing Climate Center	320 × 160	Wu et al. (2019)
EC‐Earth3	Europe	EC‐Earth Consortium	512 × 256	Doscher et al. (2022)
FGOALS‐g3	China	Chinese Academy of Sciences	180 × 80	Tang et al. (2019)
IPSL‐CM6A‐LR	France	Institute Pierre Simon Laplace	144 × 143	Bonnet et al. (2020)
MIROC6	Japan	Atmosphere and Ocean Research Institute, The University of Tokyo	256 × 128	Tatebe, Ogura, and Nitta (2019)
MPI‐ESM1‐2‐LR	Germany	Max Planck Institute for Meteorology, Alfred Wegener Institute	192 × 96	Müller, Jungclaus, and Mauritsen (2018)
MRI‐ESM2‐0	Japan	Meteorological Research Institute	320 × 160	Yukimoto, Kawai, and Koshiro (2019)
NorESM2‐MM	Norway	NorESM Climate modeling Consortium	288 × 192	Seland, Bentsen, and Olivié (2020)

Terrain data, comprising elevation (*dem*), slope (*slop*), and aspect (*asp*), were sourced from the National Tibetan Plateau Data Center (https://data.tpdc.ac.cn/). Concurrently, soil data, including soil pH (*ph*), organic carbon content (*soc*), and soil type (*st*), were derived from the China High Resolution National Soil Information Grid Basic Attribute Dataset of the National Earth System Science Data Center (http://www.geodata.cn/). Both the terrain and soil datasets possessed a horizontal spatial resolution of 1 × 1 km.

### 2.2 Screening of Environmental Factors

Based on previous studies on the phenology and climate zoning of wine grapes (Zhang et al. 2016; Yue et al. 2021; Ma et al. 2023), this study identified a total of 21 environmental factors with biological significance (Table 2), encompassing heat resources, water conditions, and the ecological environment. Within this framework, heat resources and water conditions were classified as meteorological variables susceptible to alteration due to climate change. In contrast, ecological environmental factors, such as the terrain and solid data, were presumed to be invariant.

**TABLE 2:** Potential environmental variables affecting planting distribution of wine grape.

Category	Environmental variables	Abbreviation	Unit
Heat resources	≥ 10°C accumulated temperature	*at*	°C
	Frost‐free period	*ffp*	d
	Near‐surface air temperature in January	*tas*1	°C
	Daily minimum near‐surface air temperature	*tasmin*	°C
	Near‐surface air temperature in April	*tas*4	°C
	Near‐surface air temperature in May	*tas*5	°C
	Near‐surface air temperature in July	*tas*7	°C
	Near‐surface air temperature from July to September	*tas*79	°C
	Near‐surface air temperature	*tas*	°C
Water conditions	Precipitation in April	*pr*4	mm
	Precipitation in May	*pr*5	mm
	Precipitation from July to September	*pr*79	mm
	Precipitation	*pr*	mm
	Dryness index	*di*	—
	Hydrothermic coefficient	*k*	—
Ecological environment	Digital elevation model	*dem*	m
	Slop	*slop*	degree
	Aspect	*asp*	—
	Soil pH	*ph*	—
	Soil organic carbon	*soc*	—
	Soil type	*st*	—

The ≥ 10°C accumulated temperature (*at*) and the annual average near‐surface air temperature (*tas*) denotes the heat required for grape growth and development. The frost‐free period (*ffp*) reflects the required growth days. The near‐surface air temperature in January (*tas*1) and the daily minimum near‐surface air temperature (*tasmin*) are indicative of the overwintering safety and the dormancy period of grapes. The near‐surface air temperature in July (*tas*7) is reflective of the influence of high temperature on grape growth. Precipitation (*pr*) represents the hydrological input of the region. The hydrothermic coefficient (*k*) from July to September and the dryness index (*di*) of the growing season (April to September) reflect the water and heat balance of a region. The near‐surface air temperature in May (*tas*5) and the near‐surface air temperature from July to September (*tas*79) reflect the climatic impact on critical phenological phases of grape growth. Elevation (*dem*), slope (*slop*), and aspect (*asp*) are indicative of the topographical influence, while soil pH (*ph*), soil organic carbon (*soc*), and soil type (*st*) reflect the impact of soil properties on grape cultivation.

### 2.3 Calculation and Processing of Environmental Variables

The ≥ 10°C accumulated temperature (*at*) was determined using the 5‐day moving average method. The frost‐free period (*ffp*) was defined as the interval between the final and initial occurrence of 0°C each year. The hydrothermal coefficient (*K*), calculated for the months of July to September, was detailed in Equations (1) (Jin, Zuo, and Jiang 2022; Xu et al. 2023).

(1) $$K=\sum P/\left(0.1\cdot \sum T_{a}\right)$$

∑*T*_*a*_ denotes the active accumulated temperature, which was the sum of daily average temperatures ≥ 10°C from July to September. ∑*P* denotes the precipitation from July to September (*pr*79).

Dryness index (*DI*) is defined as the ratio of evapotranspiration in growing season to rainfall in the same period, as depicted in Equations (2) (Li et al. 2007).

(2) $$\begin{array}{l} \mathrm{DI}={\mathrm{ET}}_{c}/P\\ {\mathrm{ET}}_{c}=K_{c}\times {\mathrm{ET}}_{0} \end{array}$$

*ET*_*c*_ represents the evapotranspiration during grape‐growing season (April–September). *P* signifies the precipitation over the same period. *K*_*c*_ denotes the crop coefficient, which is assigned a value of 0.8. *ET*_0_ denotes the reference crop evapotranspiration, as illustrated in Equations (3).

(3) $${\mathrm{ET}}_{0}=\frac{0.408\Delta \left(R_{n}-G\right)+\gamma \frac{900}{T+273}u_{2}\left(e_{s}-e_{a}\right)}{\Delta +\gamma \left(1+0.34u_{2}\right)}$$

*R*_*n*_ expressed in Equations (4) denotes the net radiation of crop surface (MJ·m^−2^·d^−1^). *G* expressed in Equations (5) denotes the soil heat flux. *U*_2_ denotes the average wind speed at 2 m height (m/s) (Chen et al. 2016). *Es* denotes the saturated water vapor pressure (kPa). *E*_*a*_ expressed in Equations (6) denotes the actual water vapor pressure (kPa). Δ expressed in Equations (7) denotes the slope (kPa·°C^−1^) on the curve of saturated water vapor pressure and temperature, as shown in Equations (7). *γ* expressed in Equations (8) denotes the dry and wet meter constant (kPa·°C^−1^). *T* denotes the average temperature at 2 m height (°C).

(4) $$R_{n}=\mathrm{ssr}+\mathrm{str}=\left(\text{rsds}-\text{rsus}\right)-\left(\text{rlus}-\text{rlds}\right)$$

*ssr* denotes the net solar radiation on the ground. *str* denotes the net thermal radiation on the ground. *rsds* denotes the downward shortwave radiation from the earth's surface. *rsus* denotes the upward shortwave radiation from the earth's surface. *rlus* denotes the upward long wave radiation from the surface. *rlds* denotes the downward long wave radiation from the earth's surface. The units of the above variables are MJ·m^−2^·d^−1^.

(5) $$G=0.1\left[T_{i}-\left(T_{i-1}+T_{i-2}+T_{i-3}\right)/3\right]$$

*T*_*i*_ denotes the average temperature at 2 m height on the i day (°C).

(6) $$\begin{array}{l} e_{0}\left(T\right)=0.6108\exp\left(\frac{17.27\cdot T}{T+237.3}\right)\\ e_{s}=\frac{e_{0}\left(T_{\max}\right)+e_{0}\left(T_{\min}\right)}{2}\\ e_{a}=\frac{\text{hurs}\cdot e_{s}}{100} \end{array}$$

*T*_max_ denotes the daily maximum temperature (°C). *T*_min_ denotes the daily minimum temperature (°C). *hurs* denotes the relative humidity of the ground.

(7) $$\Delta =\frac{4098\times 0.6108\cdot \exp\left(\frac{17.27T}{T+237.3}\right)}{{\left(T+237.3\right)}^{2}}$$

(8) $$\gamma =\frac{c_{p}P}{\varepsilon \lambda }=0.665\cdot P$$

*P* denotes the ground pressure (kPa). *c*_*p*_ denotes the specific heat of air. *ε* denotes the ratio constant of water vapor and dry air molecules. *λ* denotes the latent heat of vaporization of water.

We calculated the annual average value of each environmental factor from 1980 to 2014, and interpolated them by downscaling method (1 km × 1 km). The accumulated temperature, average temperature, extreme temperature, frost‐free period, dryness and hydrothermal coefficient were interpolated by trend surface and residual correction method. Firstly, we established a multiple regression model for heat factors with longitude, latitude, and altitude and calculated the residual values of each factor. Secondly, based on DEM and coordinate data, spatial distribution maps of various heat factors were obtained, and the residual distribution maps were interpolated using the inverse distance weighting method. Finally, the spatial distribution map of heat factor was overlaid with the residual distribution map to obtain a corrected grid map (Wang, Fang, and Xiong 2023). Referring to previous research on precipitation factors (He, Guo, and Xiao 2005; Zhu et al. 2020), we used the coKrijing method to interpolate a grid map using DEM as a covariate.

All environmental data were uniformly downscaled to resolution of 30 s (about 1 × 1 km).

### 2.4 Setting and Accuracy Evaluation of MaxEnt Model

The MaxEnt model, accessed from its official website (https://biodiversityinformatics.amnh.org/open_source/maxent/), offers two parameters to optimize the accuracy: the regularization multiplier (RM) and feature combinations (FCs). The FCs parameter includes linear (L), quadratic (Q), hinge (H), product (P), and threshold (T) options. Drawing on precedent studies (Yan et al. 2021; Fu et al. 2023), the RM was set with an initial step of 0.5, with a range from 0.5 to 4. The FCs were configured as L, LQ, H, LQH, LQHP, and LQHPT, respectively. Consequently, 48 distinct combinations of RM and FCs were formulated, and the combination with the lowest AICc value was identified as the optimal model.

According to the presence probability (P) estimates derived from the model, the Jenks natural breaks classification method was applied to delineate the suitability classes for the areas as follows: the unsuitable area (0 ≤ *p* < 0.05), the lowly suitable area (0.05 ≤ *p* < 0.33), the moderately suitable area (0.33 ≤ *p* < 0.66), and the highly suitable area (0.66 ≤ *p* < 1) (Fu et al. 2024).

## 3 Results

### 3.1 Assessment of Climate Models

**FIGURE 2:**
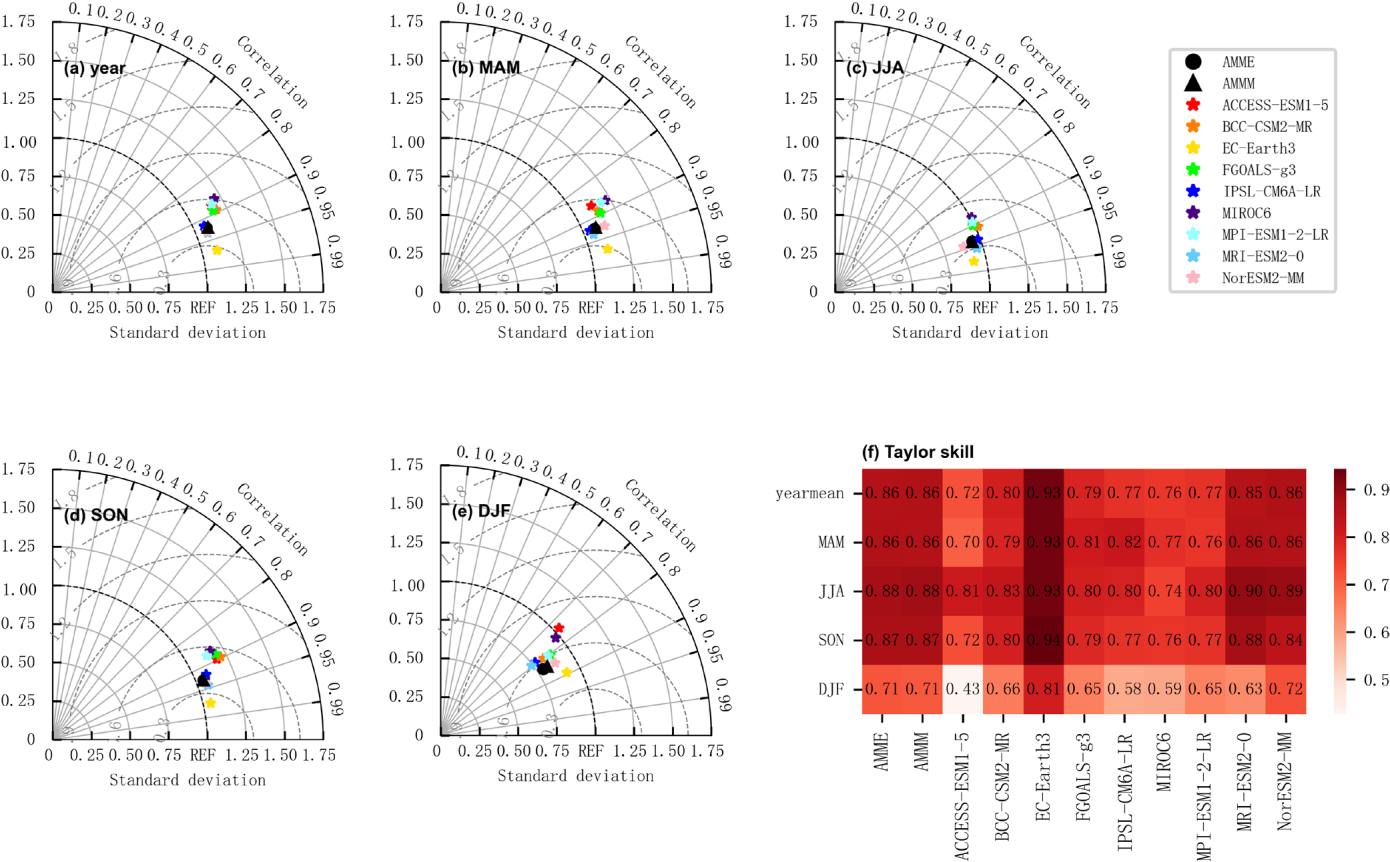
Taylor diagrams and Taylor scores for the average temperature between CMIP6 models and observations in the northwest region of China from 1980 to 2014: (a) annual average, (b) spring, (c) summer, (d) autumn, (e) winter, (f) Taylor score.

**FIGURE 3:**
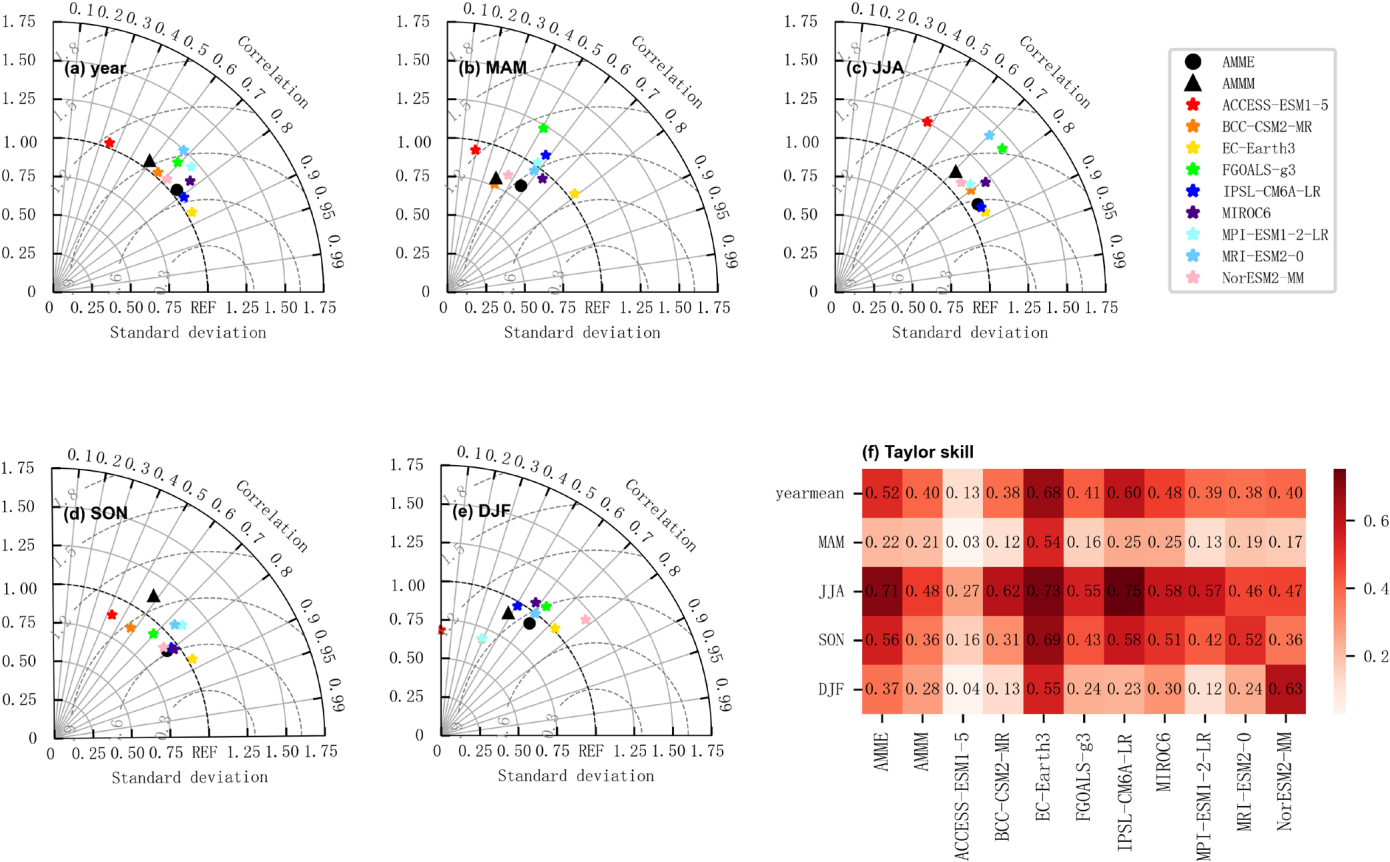
Taylor diagrams and Taylor scores for the precipitation between CMIP6 models and observations in the northwest region of China from 1980 to 2014: (a) annual average, (b) spring, (c) summer, (d) autumn, (e) winter, (f) Taylor score.

Figures 2 and 3 presented the Taylor diagrams and Taylor scores comparing the average temperature and precipitation between nine CMIP6 models and observational data in the Northwest China from 1980 to 2014. The azimuth of the model point represented the correlation coefficient between the model and the observational data, the radial distance from the origin to the model point signifies the standard deviation, and the concentric circles centered at the reference point ref. denote the normalized root mean square error (*RMSE*). Results indicated that the nine CMIP6 models have better simulation effect on temperature than precipitation. Specifically, in terms of temperature, all models exhibited robust performance, with the exception of winter simulations. Among the models, EC‐Earth3 demonstrated the best simulation across all seasons and for the annual mean, with correlation coefficients exceeding 0.95 for annual spring, summer and autumn means, and slightly below 0.9 for winter. The average annual value and normalized *RMSE* of spring, summer and autumn were all below 0.3, while winter values were below 0.6. The average annual value and Taylor scores for spring, summer and autumn were above 0.9, and above 0.8 for winter. Regarding precipitation, the simulation accuracy was comparatively lower across all models, but EC‐Earth3 still outperformed the others. The correlation coefficient between the average annual value and each season was about 0.8, the standard deviation was around 1, the *RMSE* was about 0.6, and the Taylor score was above 0.5. Collectively, the EC‐Earth3 model showed certain advantages in simulating the climate of Northwest China, leading to its selection for future climate scenarios.

### 3.2 Variable Selection and Model Accuracy

**FIGURE 4:**
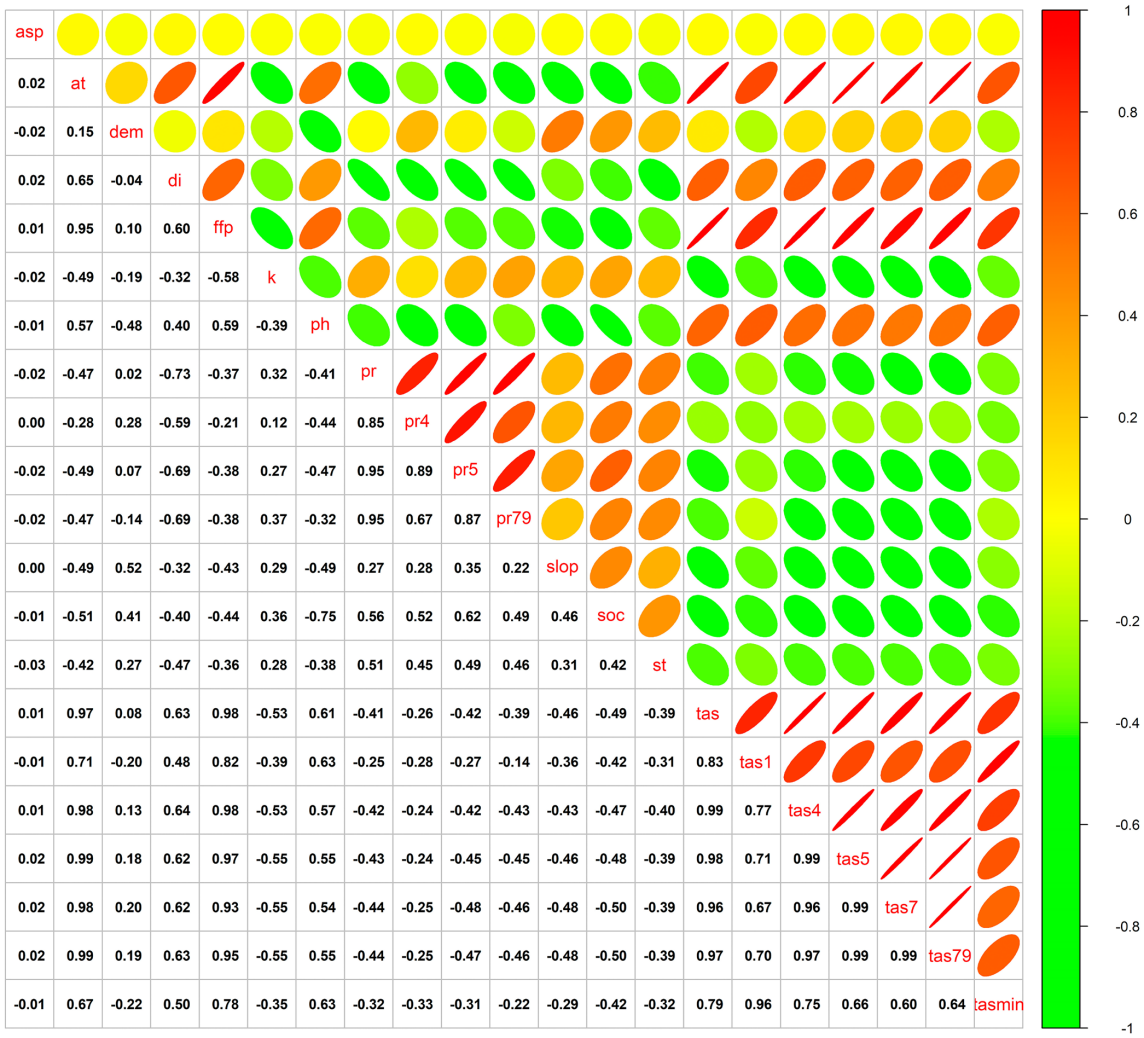
Correlation of each environmental variables.

**FIGURE 5:**
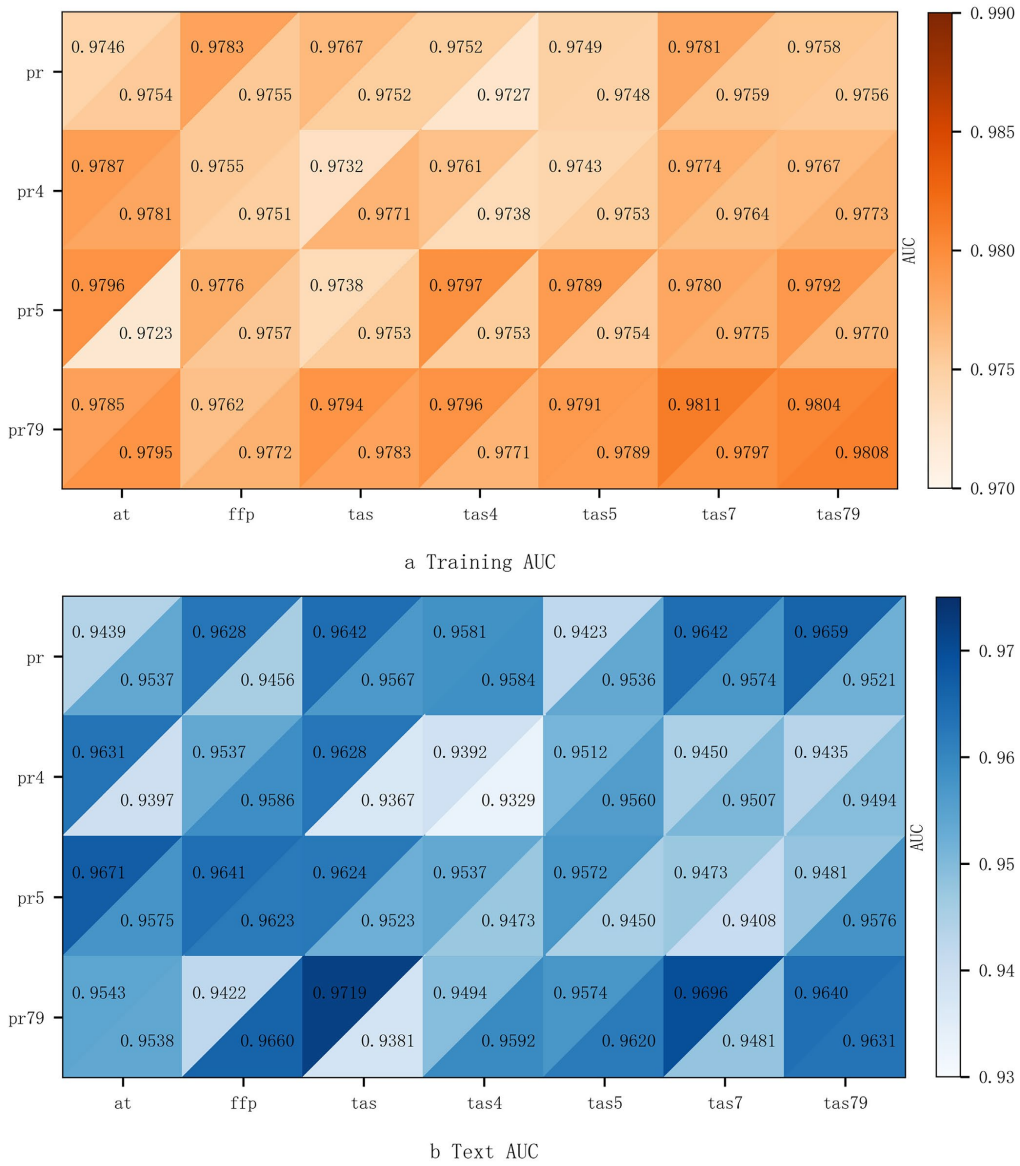
Areas under the receiver operating characteristic curve (AUC) of MaxEnt model under 12 combinations of environment variables, where the upper left cell indicates January temperature (*tas*1), and the lower right cell indicates extreme temperature (*tasmin*).

High correlations among environmental variables can influence the simulation outcomes. To mitigate this impact, the variables were categorized according to their correlation coefficients, and a combination with high predictive accuracy was selected for further analysis. Figure 4 showed that the correlation coefficient between the ≥ 10°C accumulated temperature (*at*), the frost‐free period (*ffp*), the near‐surface air temperature (*tas*), the near‐surface air temperature in April (*tas*4), the near‐surface air temperature in May (*tas*5), the near‐surface air temperature in July (*tas*7), and the near‐surface air temperature from July to September (*tas*79) were high. The correlation coefficient between the precipitation (*pr*), the precipitation in April (*pr*4), the precipitation in May (*pr*5), and the precipitation from July to September (*pr*79) were high. The correlation coefficient between the near‐surface air temperature in January (*tas*1) and the daily minimum near‐surface air temperature (*tasmin*) were high. Accordingly, a total of 56 variable combinations were obtained and the models were established respectively. Figure 5 demonstrated that the training and test AUC of all models surpassed 0.9, signifying robust performance. The optimal results were attributed to the near‐surface air temperature in July (*tas*7), the precipitation from July to September (*pr*79), and the near‐surface air temperature in January (*tas*1), with training and test AUC of 0.9811 and 0.9696, respectively.

### 3.3 Changes in Suitable Areas and Key Environmental Variables

**FIGURE 6:**
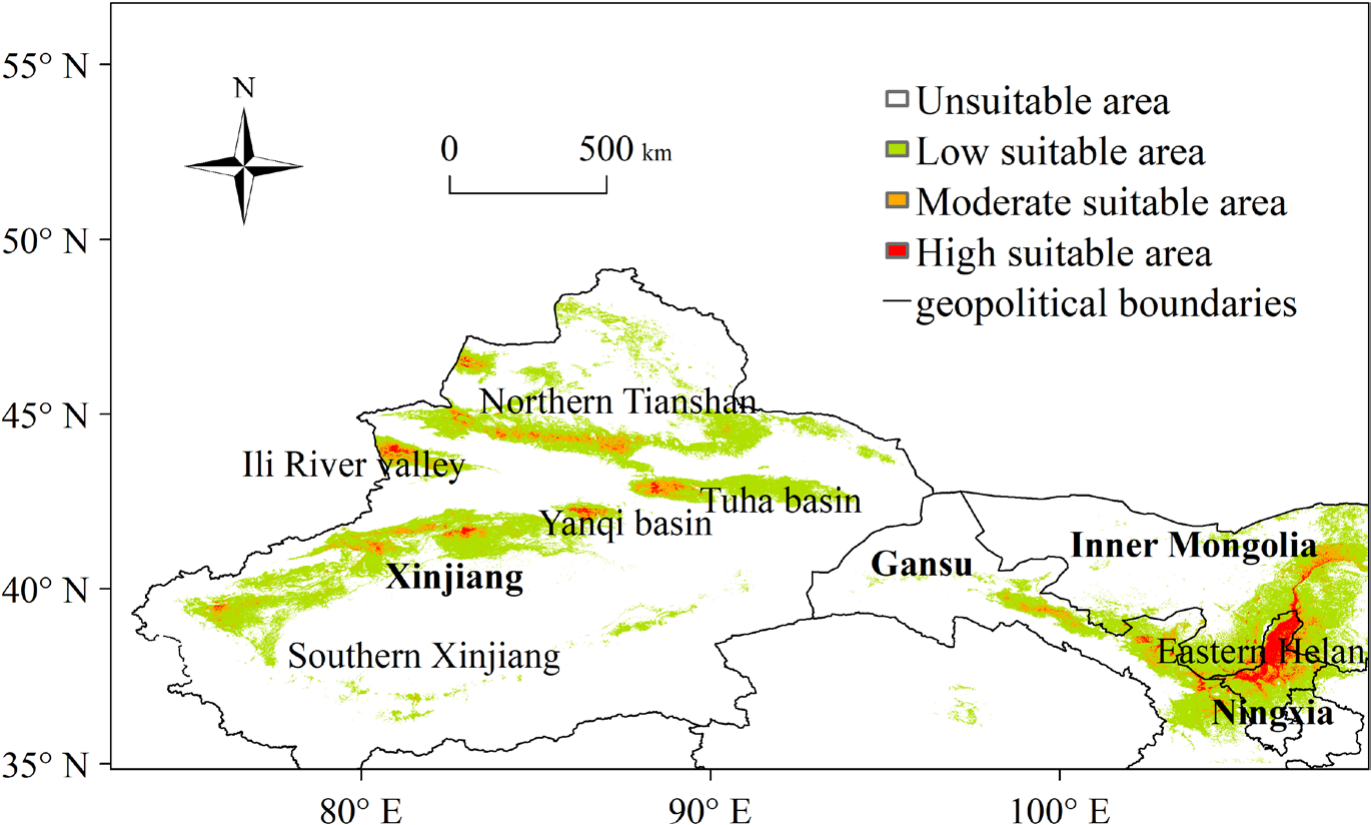
Northwestern region climatic suitability area for wine grapes.

Figure 6 illustrated the simulation results for the wine grapes suitable areas in the northwest region from 1980 to 2014. In Ningxia, the highly suitable areas were predominantly located in Shizuishan, Helan, Xixia, Yongning, Qingtongxia, and Hongsibao on the eastern foothills of Helan Mountain. In Inner Mongolia, the highly suitable areas were found along the Yellow River in the Linhe area of Wuhai and Bayan Zhuoer. In Gansu, the highly suitable areas were aligned with the Qilian Mountains in Lanzhou, Wuwei, Jinchang, Zhangye, Jiuquan, and Jiayuguan. In Xinjiang, the highly suitable areas were concentrated in the Ili River Valley in western Xinjiang, as well as in Urumqi, Changji, Bole, and Tacheng areas at the northern foot of the Tianshan Mountains in northern Xinjiang, the Yanqi Basin, Tuha Basin in eastern Xinjiang, and the Aksu, Muzart, and Kashgar areas at the southern foot of the Tianshan Mountains in southern Xinjiang, showing the characteristics of distribution along river oases.

In terms of percent contribution rate, the precipitation from July to September (*pr*79), soil pH (*ph*), and elevation (*dem*) were identified as having a significant influence on the model. Concurrently, the precipitation from July to September (*pr*79), the dryness index (*di*), and the near‐surface air temperature in July (*tas*7) emerged as the three most influential factors. Analysis of permutation importance revealed that the precipitation from July to September (*pr*79), elevation (*dem*), and the near‐surface air temperature in January (*tas*1) were the most significant, suggesting a higher dependency of the model on these variables. Focusing on climate factors, the precipitation from July to September (*pr*79) exhibited a high percent contribution and permutation importance, signifying its pivotal role in influencing the distribution of wine grapes. Although the near‐surface air temperature in January (tas1) had a moderate percentage contribution, its high permutation importance indicated that it acts as a critical limiting factor.

Similarly, the Jackknife test elucidated the individual contributions of various potential factors to the model's predictive performance. From the perspective of one variable only, the near‐surface air temperature in July (*tas*7), the precipitation from July to September (*pr*79), the hydrothermic coefficient (*k*) and the dryness index (*di*) demonstrated higher scores, highlighting their importance in model accuracy. When considering the omission of any single variable, the model's performance remained above 0.9, indicating that no individual factor is decisive in governing the potential distribution of wine grapes (Table 3).

**TABLE 3:** Importance of environmental variables of wine grape.

Environmental variables	Percent contribution	Permutation importance	With only variable	Without variable
*tas*1	4.8	10.2	0.75	0.94
*tas*7	5.6	4.4	0.86	0.95
*pr*79	24.2	26.5	0.82	0.93
*k*	0.8	9.0	0.82	0.94
*di*	9.4	5.6	0.81	0.94
*dem*	11.3	21.4	0.76	0.93
*slop*	4.9	2.6	0.67	0.94
*asp*	4.6	1.7	0.60	0.95
*ph*	22.1	8.4	0.80	0.94
*soc*	4.6	7.9	0.75	0.95
*st*	7.7	2.3	0.69	0.95

**FIGURE 7:**
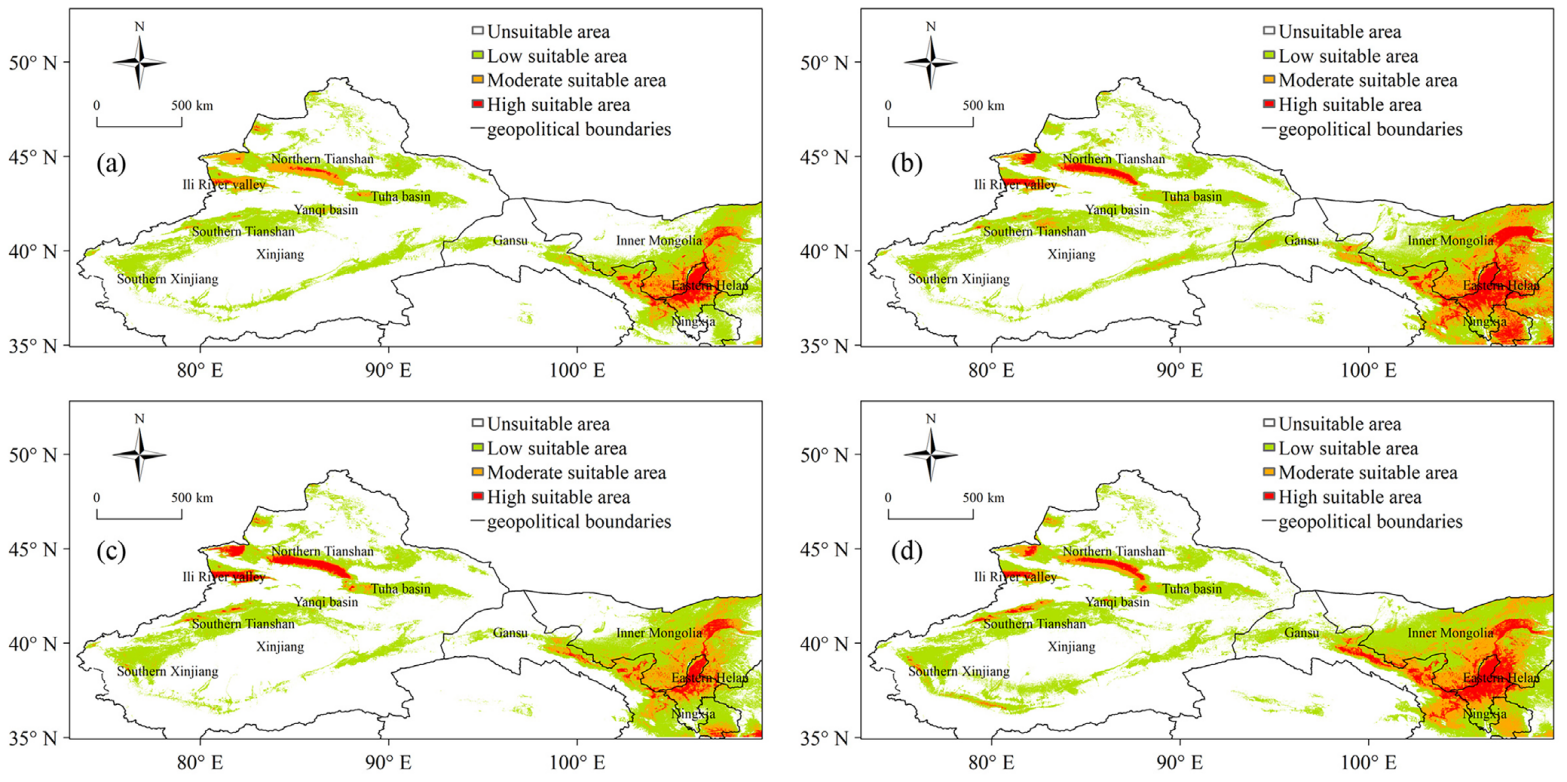
Climatic suitability areas for wine grapes in the Northwestern region under different scenarios: (a) SSP245 in the next 30 years, (b) SSP245 in the next 50 years, (c) SSP585 in the next 30 years, (d) SSP585 in the next 50 years.

Figure 7 depicted the projected suitable areas for wine grapes in Northwest China during the periods 2035 ~ 2065 and 2055 ~ 2085 under SSP245 and SSP585 scenarios. Comparatively, the highly suitable areas in 2035 ~ 2065 were similar to historical period but exhibited a tendency to expand, particularly around the eastern foothills of Helan Mountains. Specifically, the expansion would extend westward along the Yellow River to the Zhongwei area, southward along the Qingshui River to the Tongxin area, eastward to the Lingyan Plateau and toward the Yanchi area. In Inner Mongolia, the highly suitable areas were projected to extend from the Linhe area of Bayan Zhuoer across entire Houtao Plain. In the Wuwei area of Gansu Province, the highly suitable areas were expected to develop along the Shiyang River to the Shiyang River oasis area. In Xinjiang, the highly suitable areas in the northern foothills of the Tianshan Mountains and the Ili River Valley showed an increasing trend, with a notable emergence of high suitability under the SSP585 scenario in the northern foothills of the Tianshan Mountains.

Under two scenarios in 2055 ~ 2085 period, the highly suitable areas for wine grapes were anticipated to experience further expansion. Under SSP245, the highly suitable areas in Ningxia were projected to extend towards the Guyuan and Pingliang regions, reaching the Ordos Plateau and Maowusu Sandy Land towards the east. In Xinjiang, new areas of high suitability were expected to emerge in the Tuha Basin. Under SSP585, the highly suitable areas were predicted to expand into the Yanqi Basin in eastern Xinjiang and Kashgar and Hotan areas in southern Xinjiang, although a reduction was expected in the northern foothills of the Tianshan Mountains. Overall, the highly suitable areas would grown more under the SSP245 scenario.

### 3.4 Spatial Distribution and Impact of Key Environmental Factors

**FIGURE 8:**
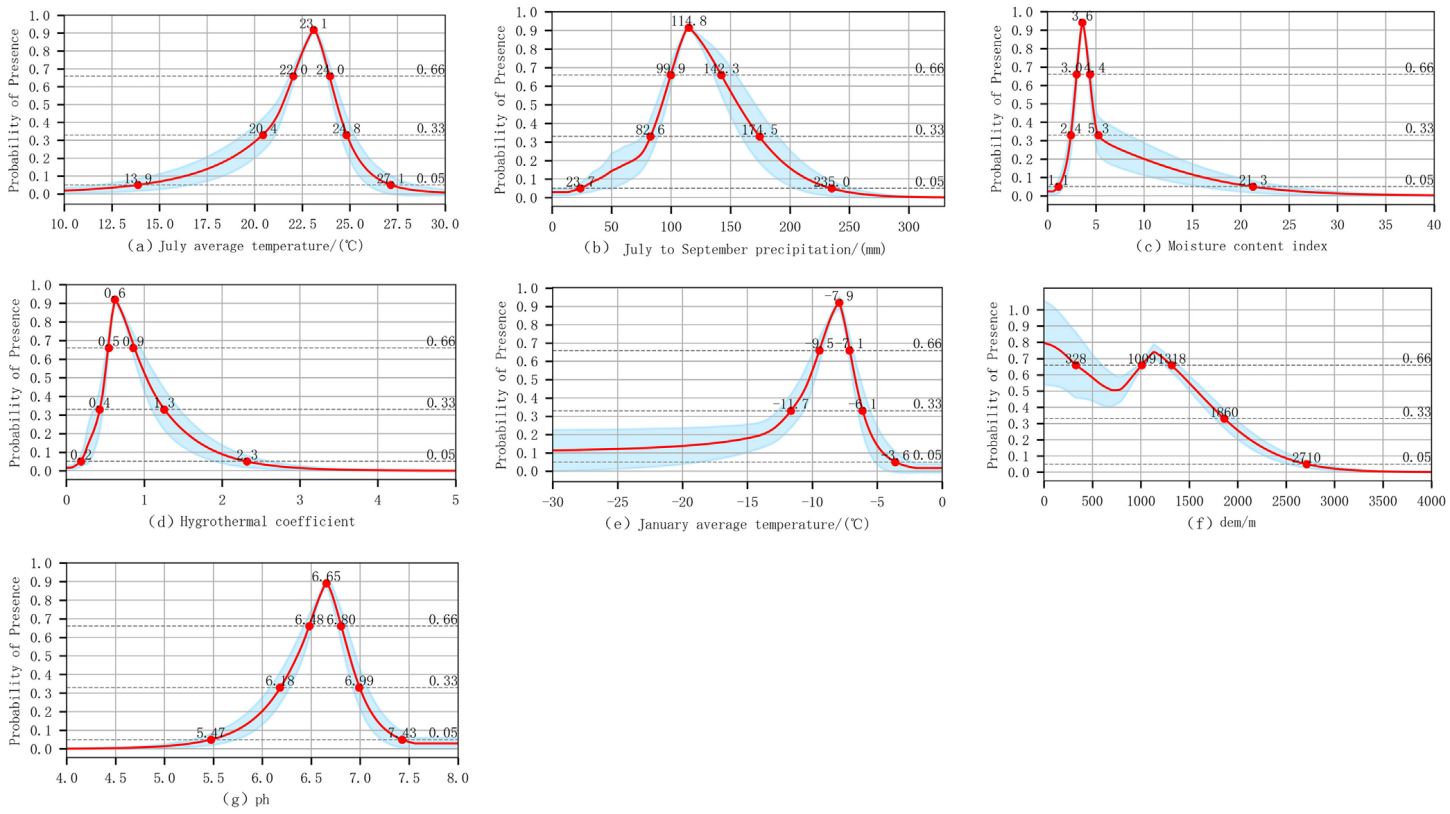
Response curves of climate‐related environmental variables to model prediction.

Figure 8 presented the response curves of climatic factors. The presence probability (*p*) was utilized to delineate suitability, with *p* ≥ 0.66 designating a region as highly suitable area and *p* ≥ 0.05 as cultivable. Within the highly suitable area, the thresholds were as follows: for the near‐surface air temperature in July (*tas*7), the range was 22.0°C ~ 24.0°C; for the precipitation from July to September (*pr*79), the range was 99.9 ~ 142.3 mm; for the dryness index (*di*), the range was 3.0 ~ 4.0; for the hydrothermic coefficient (*k*), the range was 0.5 ~ 0.9; for the near‐surface air temperature in January (*tas*1), the range was −9.5°C ~ −7.1°C; for the elevation (*dem*), the range was < 328 m or 1009 ~ 1318 m; for the pH (*ph*), the range was 6.48 ~ 6.8. Within the cultivable area, the threshold for the respective climatic factors were as follows: for the near‐surface air temperature in July (*tas*7), the range was 13.9°C ~ 27.1°C; for the precipitation from July to September (*pr*79), the range was 23.7 ~ 235 mm; for the dryness index (*di*), the range was 1.0 ~ 21.3; for the hydrothermic coefficient (*k*), the range was 0.2 ~ 2.3; for the near‐surface air temperature in January (*tas*1), the range was ≤ −3.6°C; for the altitude (*dem*) the range was 328 ~ 1009 m or 1318 ~ 1860 m; for the pH (*ph*), the range was 5.47 ~ 6.18 or 6.99 ~ 7.43.

**FIGURE 9:**
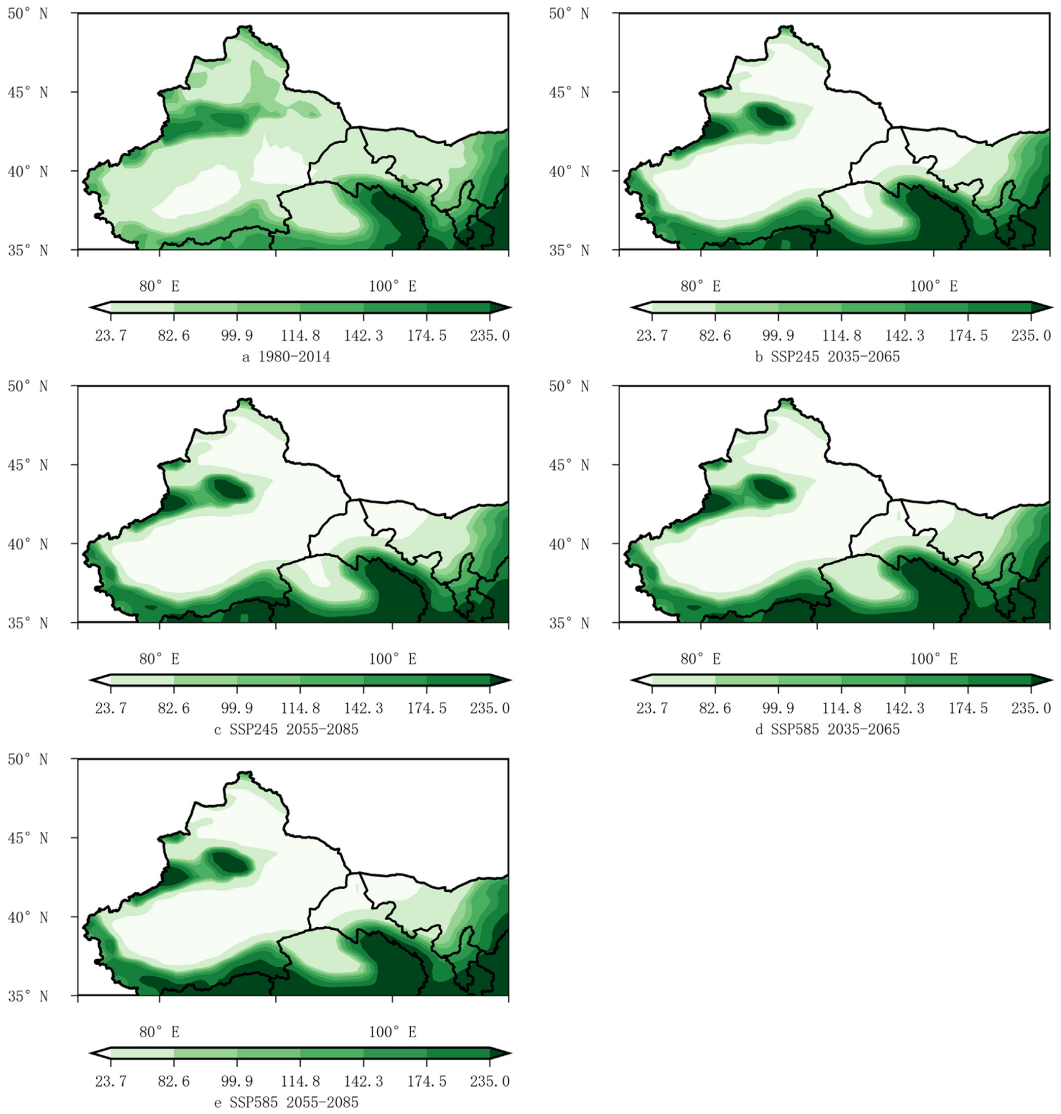
Spatial distribution maps of the precipitation from July to September (*pr*79) in the Northwestern region for 1980 ~ 2014 and under different scenarios for 2035 ~ 2065 and 2055 ~ 2085.

**FIGURE 10:**
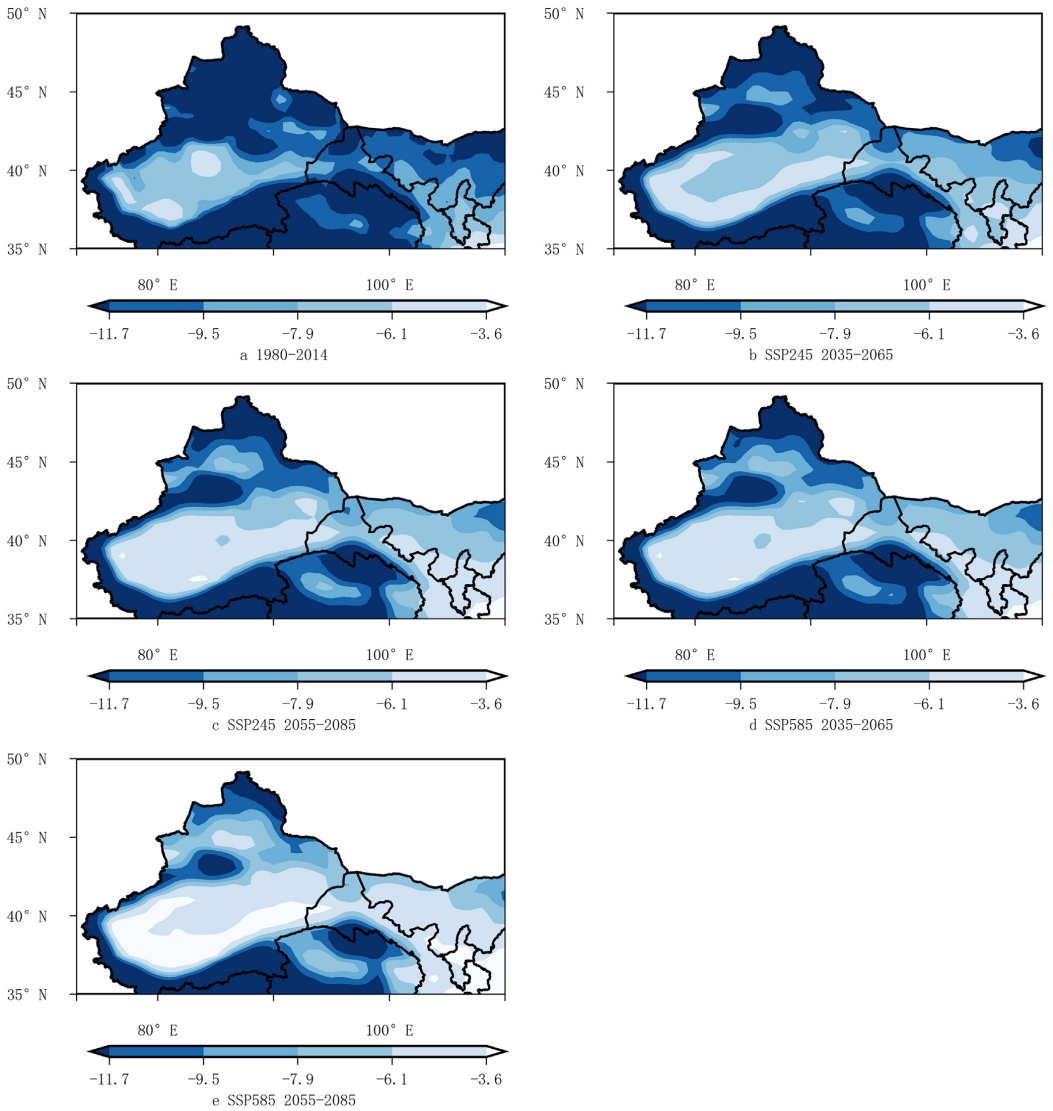
Spatial distribution maps of the near‐surface air temperature in January (*tas*1) in the Northwestern region for 1980 ~ 2014 and under different scenarios for 2035 ~ 2065 and 2055 ~ 2085.

**FIGURE 11:**
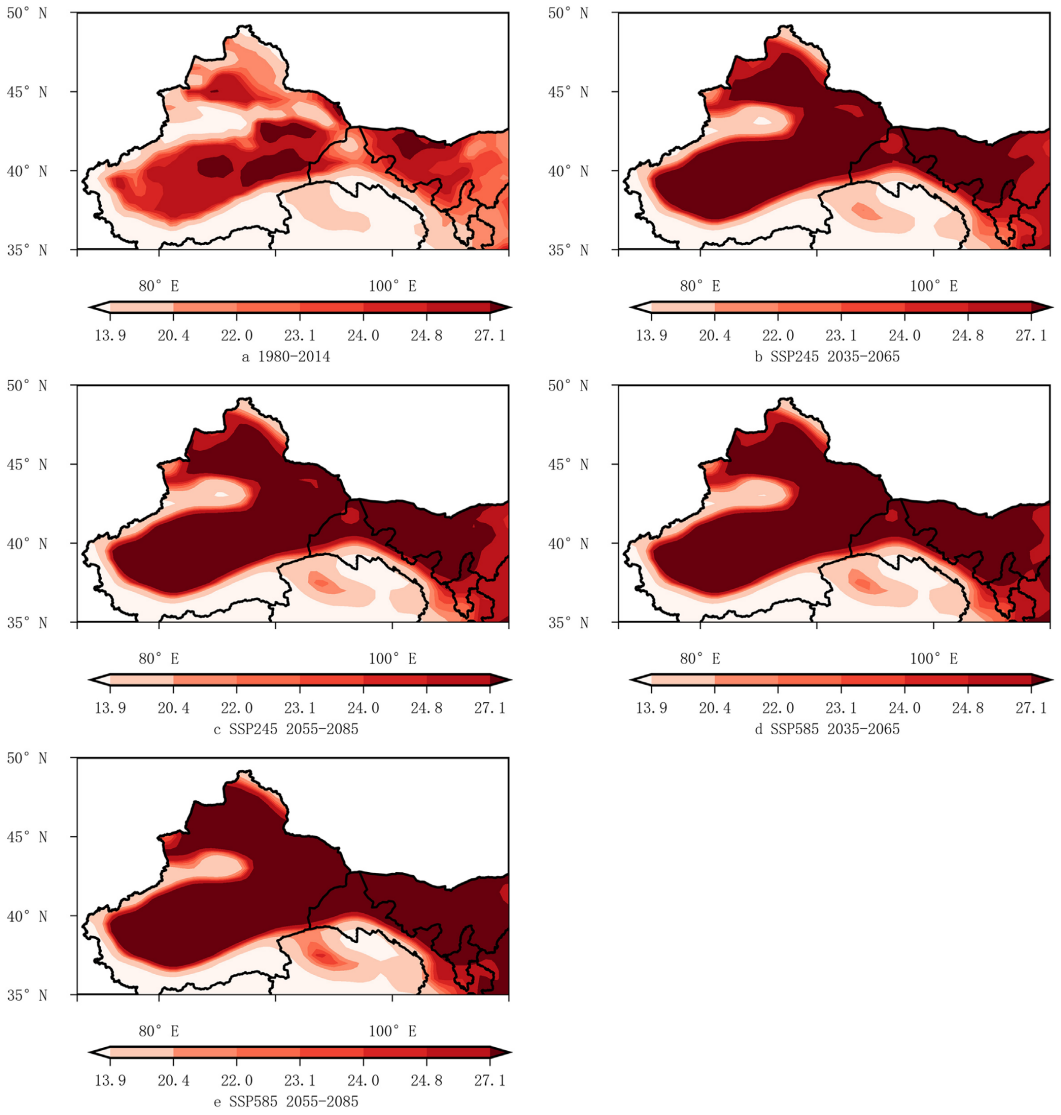
Spatial distribution maps of the near‐surface air temperature in July (*tas*7) in the Northwestern region for 1980 ~ 2014 and under different scenarios for 2035 ~ 2065 and 2055 ~ 2085.

Figures 9, 10, 11 showed the spatial distribution of the precipitation from July to September (*pr*79), the near‐surface air temperature in January (*tas*1) and the near‐surface air temperature in July (*tas*7) during historical periods (1980–2014) and future periods (2035–2065 and 2055–2085). The precipitation from July to September (*pr*79) was an important environmental factor affecting the suitable areas of wine grapes in the northwest region. Compared with the historical period, there would be a large area reduce of the precipitation from July to September (*pr*79) in the Tarim Basin‐East Xinjiang‐Junggar Basin, which may affect the suitable areas of wine grapes in the Yanqi Basin and Tuha Basin. However, the spatial distribution changes of the precipitation from July to September (*pr*79) were not too great in the eastern foothills of Helan Mountain in Ningxia, the northern and southern foothills of Tianshan Mountains, southern Xinjiang, and the Inner Mongolia region of Gansu (Figure 9).

Relative to historical periods, the near‐surface air temperature in January (*tas*1) exhibited an increasing trend, particularly in the eastern foothills of Helan Mountains and southern Xinjiang, which would provide more safe wintering environments for wine grapes. However, excessive warming, such as in the SSP585 scenario during 2055–2085, a significant number of areas in Southern Ningxia were projected to experience temperatures ≥ −3.6°C, potentially impacting the dormancy period of wine grapes (Figure 10).

The near‐surface air temperature in July (*tas*7) showed a significant increase in the future under simulation. In comparison with historical period, under the SSP245 scenario during 2035–2065, significant warming (*tas*7 ≥ 27.1°C) was anticipated to extend from the Tarim Basin, East Xinjiang, the Junggar Basin to Western Inner Mongolia. The spatial distribution of *tas*7 under SSP245 during 2055–2085 was similar to that under SSP585 during 2035–2065, with high temperatures further expanding to the eastern foothills of Helan Mountains. Under SSP585 during 2055–2085, the prevalence of high temperatures was expected to persist across Northern Ningxia and extend westward into Western Shaanxi. The high near‐surface air temperature in July can cause high‐temperature disasters, impacting the growth and development of wine grapes, which may account for the more pronounced contraction in highly suitable area under SSP585 during 2055–2085 in the northern foothills of the Tianshan Mountains (Figure 11).

## 4 Discussion

By comparison with the CN05.1 grid observational dataset spanning from 1980 to 2014, this study assessed the performance of 9 CMIP6 models in simulating the spatial distribution and interannual variability of temperature and precipitation in Northwest China. Results showed that EC‐Earth3 performed well in simulating temperature, precipitation, and interannual changes. Collectively, the models' performance in simulating temperature exceeded that of precipitation, with winter simulations lagging behind those of other seasons, a finding consistent with prior research (Yang et al. 2021). The errors in climate model predictions may lead to inaccurate simulation of future changes in wine grape suitable areas. For instance, the underestimation of precipitation from July to September (*pr*79) in the Tuha Basin has led to the omission of highly suitable areas in future projections. To enhance the predictive accuracy, it may be imperative to incorporate additional high‐resolution models and employ more sophisticated downscaling techniques for environmental variables. Our investigation utilized the MaxEnt model to identify key determinants impacting wine grape suitable areas. The analysis revealed that the models containing the precipitation from July to September (*pr*79) exhibited superior performance in both modeling and validation phases, with high percent contributions, permutation importance, and single‐factor AUC contributions attributed to the precipitation from July to September (*pr*79) were high. These foundings indicated that *pr*79 (i.e., precipitation during veraison phases) was a significant factor influencing the distribution of wine grapes suitable areas in the northwest region. Prior studies have arrived at analogous conclusions. For instance, Shang et al.'s (2023) research determined that the water requirement for wine grapes during their growth period in the eastern foot of Helan Mountains ranged from 458.04–546.74 mm, with the minimum demand occurring in April and the maximum in July. Meng et al.'s (2023) experimental findings revealed that increased precipitation during the veraison phases affects grape berry ripening, leading to a reduction in phenolic content within the fruit skin. However, future alterations in the precipitation from July to September (*pr*79) in the northwest region under SSP245 and SSP585 scenarios, as projected by the EC‐Earth3 model, were not markedly divergent, suggesting that there may be no substantial shifts in the distribution pattern of suitable areas.

The permutation importance of the near‐surface air temperature in January (*tas*1) was notably high while its percent contribution percentage was relatively low, indicating that *tas*1 was a significant limiting factor. Simulation conducted by the EC‐Earth3 indicated a general increase in *tas*1 across the northwest region, potentially offering a more favorable wintering environment for wine grapes and contributing to the expansion of suitable areas. However, the dormancy period of grapes necessitates a certain level of low‐temperature accumulation, therefore, an excessive increase in *tas*1 may disrupt this period, consequently impacting the grapes' suitable range. Under SSP585 scenario during 2055 ~ 2085 period, *tas*1 was projected to rise significantly increase in the southern region of Ningxia, which could diminish the suitability of wine grapes in this region. The near‐surface air temperature in July (*tas*7) not only characterizes the impact of high‐temperature heat damage on grape cultivation, but also represents the heat resources required for grape growth. Our results revealed that the near‐surface air temperature in July (*tas*7) did not exhibit high percent contribution or permutation importance. However, in other models, accumulated temperature variables demonstrated superior performance in terms of percent contribution. This suggested a necessity to identify a novel variable that captures the impact of high temperatures on grapes without being highly correlated with environmental factors that represent thermal resources. The EC‐Earth3 model simulations indicated substantial alterations and increases in the spatial distribution of the near‐surface air temperature in July (*tas*7), suggesting that projected higher temperatures could result in a reduction of wine grapes suitable areas. Specifically, in 2055 ~ 2085 period, the highly suitable areas for wine grapes in the northern foothills of the Tianshan Mountains were expected to experience a more pronounced reduction under the SSP585, ultimately leading to a comparatively maller expansion of highly suitable area in the northwest region under SSP585 as opposed to SSP245.

In the selection and evaluation of global climate models, particularly within the CMIP6 framework, researchers were tasked with filtering and assessing models specific to geographical regions. This process entails comparing model historical simulations (i.e., data prior to 2015) against actual observational data. Given the disparities in spatial resolution among models, which seldom align with observational data, it is imperative to standardize model data to the resolution of observational data through interpolation techniques such as bilinear interpolation to facilitate subsequent analysis. For instance, Yang et al. (2021) utilized bilinear interpolation to evaluate temperature and precipitation data from 20 models, exploring the applicability of these data across various regions in China. While dynamic downscaling has been employed in previous studies to investigate regional climates, these methods are heavily contingent upon parameterization schemes and model dynamics. This study, however, aimed to predict future shifts in the suitable areas, thus opting for a simplified downscaling approach. In selecting the most suitable climate model for the northwest region of China, our results showed that the EC‐Earth3 model offers more precis simulations than the BCC‐CSM2‐MR model, which was designed to capture regional variations of the Asian monsoon. The inland regions of Xinjiang, Ningxia, and Gansu in Northwest China are less influenced by monsoons, may explain the BCC‐CSM2‐MR model's suboptimal performance in simulating these specific areas.

Within the scope of this study, ecological environmental factors, encompassing terrain and soil characteristics, were generally perceived as invariant to climate change (Zhang and Li 2010; Yang, Mu, and Li 2012). Our simulation has demonstrated that elevation (*dem*) and soil pH (*ph*) have a significant impact on the model. While it is anticipated that terrain factors will remain constant in the near term, additional research is warranted to ascertain the potential alterations in soil‐related factors in response to climate warming over the forthcoming 30–50 years.

## 5 Conclusions

This study conducted a comparative analysis of the applicability of nine CMIP6 climate models in the northwest region of China. Utilizing the MaxEnt model, we projected the climatic suitability for wine grapes under both historical and future climate scenarios and examined the environmental factors influencing the adaptability of wine grapes. The results can provide scientific decision‐making references for the introduction, large‐scale promotion, and reasonable layout of wine grapes in the northwest region. Our findings indicated during the historical period (1980–2014), the highly suitable areas for wine grapes in the northwest region were predominantly located in the eastern foothills of Helan Mountains in Ningxia, along the Yellow River in Wuhai and Linhe of Inner Mongolia, along the Qilian Mountains in Wuwei, Zhangye and Jiayuguan of Gansu, and along rivers and oases in the northern foothills of Tianshan Mountains, Ili River Valley, Tuha Basin, Yanqi Basin, Aksu, Muzart, and Kashgar of Xinjiang. *Pr*79, soil pH, altitude, and *tas*1 were the main factors affecting the suitable areas of wine grapes. Relative to historical period (1980–2014), the highly and moderately suitable areas of wine grapes under SSP245 and S585 were projected to increase. Over the next half‐century (2055–2085), the suitable areas under SSP245 scenario were anticipated to exceed that of the SSP585 scenario.

## Author Contributions

**Yanyan Liu:** conceptualization (lead), data curation (equal), funding acquisition (lead), resources (equal), software (equal), visualization (equal), writing – original draft (equal), writing – review and editing (equal). **Xuejia Shi:** data curation (equal), formal analysis (equal), investigation (equal). **Hongjuan Du:** formal analysis (equal), investigation (equal), validation (equal). **Mengjiao Jiang:** resources (equal), software (equal), supervision (equal). **Fang Li:** formal analysis (equal), validation (equal), visualization (equal). **Jing Wang:** data curation (equal), formal analysis (equal), software (equal). **Xiaoyu Zhang:** formal analysis (equal), funding acquisition (equal), visualization (equal), writing – review and editing (equal).

## Conflicts of Interest

The authors declare no conflicts of interest.

## Data Availability

We used open‐access data from the Global Biodiversity Information Facility database (GBIF, https://www.gbif.org/) and the Earth System Grid Federation (ESGF, https://aims2.llnl.gov).
